# Atopy--a favourable prognostic factor for survival in Hodgkin's disease.

**DOI:** 10.1038/bjc.1983.176

**Published:** 1983-08

**Authors:** P. L. Amlot, J. Slaney, R. Brown

## Abstract

One hundred and forty-eight patients with Hodgkin's disease (HD) were stratified into 4 groups according to atopic status. Group 1 had a personal history of atopy and Group 2 a family history of atopy. In Groups 3 and 4 there was no history of atopy but high serum IgE levels (Group 3) and normal IgE levels (Group 4). Comparison of the survival of these groups by the logrank method showed a significant trend (P = less than 0.0001) where survival was ranked Group 1 greater than 2 greater than 3 greater than 4. Known prognostic factors in HD--age, sex, stage, symptoms and histology--had to be taken into account, since their distribution differed between the atopic groups. In Group I there was more stage IA and IIA disease and less "B" symptoms, in Group 3 more nodular sclerosis histology and more "B" symptoms and in Group 4 more lymphocyte-depleted histology and a higher mean age than expected from their distribution in the combined groups. Adjustment to allow for the variation in each of the other prognostic factors and for a combination of age, symptoms and histology still showed a significant trend of survival on the basis of atopic status. The increased survival of atopic patients suggests that atopic mechanisms or the genetic basis to atopy has a protective effect in HD either directly or by interaction with treatment.


*Br. J. Cancer* (1983), **48**, 209–215

### Atopy—A favourable prognostic factor for survival in Hodgkin's Disease

P.L. Amlot, J. Slaney & R. Brown

*Departments of Medicine and Community Medicine, Guy's Hospital Medical School, London SE1 9RT.*

**Summary** One hundred and forty-eight patients with Hodgkin's disease (HD) were stratified into 4 groups according to atopic status. Group 1 had a personal history of atopy and Group 2 a family history of atopy. In Groups 3 and 4 there was no history of atopy but high serum IgE levels (Group 3) and normal IgE levels (Group 4). Comparison of the survival of these groups by the logrank method showed a significant trend ($P = < 0.0001$) where survival was ranked Group $1 > 2 > 3 > 4$.

Known prognostic factors in HD—age, sex, stage, symptoms and histology—had to be taken into account, since their distribution differed between the atopic groups. In Group I there was more stage IA and IIA disease and less "B" symptoms, in Group 3 more nodular sclerosis histology and more "B" symptoms and in Group 4 more lymphocyte-depleted histology and a higher mean age than expected from their distribution in the combined groups. Adjustment to allow for the variation in each of the other prognostic factors and for a combination of age, symptoms and histology still showed a significant trend of survival on the basis of atopic status. The increased survival of atopic patients suggests that atopic mechanisms or the genetic basis to atopy has a protective effect in HD either directly or by interaction with treatment.

Atopy was a term introduced by Coca & Cooke (1923) to describe the syndrome of common allergic diseases—hay fever, asthma, atopic eczema and urticaria—which had a genetic basis. The conundrum exists to this day as to why this complex immune system, involving B cells producing predominantly the IgE class of antibodies and a regulatory T cell system, basophils, mast cells and eosinophils, should be maintained when it apparently turns harmless environmental antigens into allergens detrimental to the health of the host. Teleologically, the most plausible explanation is that this system evolved as a protection against helminthic infestation, although the role that antibodies of IgE class play in helminthic immunity has not clearly been established (Ogilvie & Jones, 1973). In developed countries where public health measures have all but eradicated the major helminthic parasites it is possible that this system has become redundant and its expression in hyper-reactive individuals is atopic disease.

Another role has been suggested for IgE in protective immunity against cancer. Early studies purported to show that the incidence of atopy was decreased in cancer patients (Fisherman, 1960; Mackay, 1966) but subsequent large controlled studies were unable to verify the earlier claims (McKee *et al.*, 1967; Shapiro *et al.*, 1971) and the consensus of opinion now is that the presence of atopy does not protect against oncogenesis (for review, see Rosenbaum & Dwyer, 1977). However none of these studies examined how atopic cancer patients fared compared to their non-atopic counterparts. The present study examines the effect of pre-existing atopy on the survival of patients with Hodgkin's disease (HD) as well as other parameters of disease expression. From the standpoint of allergic disease, HD is a particularly apt example of malignancy because like helminthic parasites it is associated with raised levels of serum IgE (Waldman *et al.*, 1974; Amlot & Green, 1978) and eosinophilia. The reported frequency of atopy in HD is normal (Amlot & Green, 1978; Dworin *et al.*, 1955; McCormick *et al.*, 1971) which suggests that, as with other malignant tumours, it does not protect atopic subjects from the development of HD. However, it was observed in this study that survival of atopic patients was significantly improved compared with those who were non-atopic.

### Patients and methods

### *Patients*

One hundred and forty-eight patients with Hodgkin's disease (HD) were assessed at the time of their diagnosis and prior to treatment. One

Correspondence: P.L. Amlot
Received 25 March 1983; accepted 25 May 1983.

© The Macmillan Press Ltd., 1983

hundred and twenty-seven patients were seen at Guy's Hospital between September 1972 and June 1979, while the remaining 21 were seen at the London Hospital between April 1974 and April 1977. Follow-up of survival and disease status was monitored in December 1979.

Staging of the patients accorded with the Ann Arbor system (Carbone *et al.,* 1971). Clinical staging routinely included lymphography and bone marrow trephine biopsy. Selective staging laparotomy and splenectomy were carried out in 74 cases. Prior unequivocal "B" symptoms (59 patients), contraindication to operation (8 patients) and clinical stage IA disease with lymphocyte predominance or nodular sclerosis (7 patients) were reasons for not performing staging laparotomies in the remainder.

Histology was classified according to Lukes & Butler (1966) and grouped as lymphocyte predominance (LP), nodular sclerosis (NS), mixed cellularity (MC) or lymphocyte depletion (LD).

### *Atopic symptoms*

Past or present personal histories of hay fever, perennial rhinitis, asthma, atopic eczema or urticaria and similar family histories were elicited from each patient. Questionnaires were sent to each member of the patient's direct family requesting the same information and were answered by all except 11 families. Doubtful symptoms, drug allergies, contact dermatitis and atopy evidently arising from the unaffected spouse's side of the family were disregarded. Family history extended to grandparents, parents, siblings, children, uncles, aunts and first cousins.

Patients were stratified on atopic status according to their atopic history and pre-treatment serum IgE level. Group 1 were those with a personal history of atopy; Group 2 had a family history but no personal history of atopy; Groups 3 and 4 had neither personal nor family history of atopy but had high and normal levels of serum IgE respectively.

### *Measurement of IgE*

This was performed on a pre-treatment serum sample from all patients using a double antibody radio-immuno assay (Amlot & Green, 1978). Normal IgE levels were determined from 275 healthy, non-atopic subjects whose geometric mean IgE was 15 IU ml$^{-1}$ ($\log_{10} 1.8 \pm 0.51$). The upper limit for normal IgE levels was set at 159 IU ml$^{-1}$ (antilog $\log_{10} 1.18 + 2 \times 0.51$).

Prick tests were performed on the majority of patients using a battery of common inhalant allergens described recently elsewhere (Amlot & Slaney, 1981).

### *Treatment*

The treatment policy was the same for all patients. Primary treatment for stage IA and IIA was by "mantle" or "inverted" Y fields (Kaplan & Rosenberg, 1975) to a minimum of 3,500 cGy over 4 weeks. Stage IIIA was treated by total nodal irradiation to the same dosage over 10 weeks. Patients with stages IB, IIB, IIIB, IVA and IVB were treated by combination chemotherapy predominantly with MOPP (De Vita *et al.,* 1970) and in the remainder by the MVPP variation (Nicholson *et al.,* 1970).

### *Patient survival*

Time of survival was calculated from diagnosis. Complete remission (CR) was the clinical disappearance of all disease induced by the primary treatment and which persisted for at least 3 months after the end of treatment. Patients who attained CR and subsequently relapsed or who only achieved partial or no-response but who had not died, were designated poor remission or relapse. Many such patients attained CR with further therapy.

### *Statistical analysis*

Life tables for the different groups were obtained and compared by the Logrank test (Peto *et al.,* 1977) using the London School of Hygiene and Tropical Medicine's version of the Surv-C programme (Peto *et al.,* 1977) and run at the University of London Computer Centre. Allowance was made for prognostic factors which were shown to affect survival. Statistical analysis elsewhere was by $\chi^2$ for contingency tables.

### Results

The characteristics of the groups 1–4 in respect of atopy are shown in Table I. In Group 1 the complete atopic diathesis was seen with atopic symptoms, positive prick tests and elevated IgE levels, while Group 2 shared with it an increased frequency of positive prick tests and moderately raised IgE levels having antibody activity for common inhalant allergens (shown previously by RAST) (Amlot & Slaney, 1981). Group 3 had the highest IgE levels but in this group the IgE rarely had demonstrable allergen specific antibody, (Amlot & Slaney, 1981). Group 4 showed no evidence of atopy apart from a low incidence of

**Table I** Details of atopic strata among patients with Hodgkin's disease

| | *Atopic history* | | *Prick test responses* | | | |
|---|---|---|---|---|---|---|
| *Atopic group* | *Personal* | *Family* | *No. tested* / *No. in group* | *% + ve >5 mm* | *% + ve 3–5 mm* | *Geometric mean IgE level in IU ml⁻¹* |
| 1 | + | + or − | 29/29 | 69 | 24 | 186 |
| 2 | − | + | 23/26 | 26 | 43 | 54 |
| 3 | − | − | 24/31 | 4 | 21 | 794 |
| 4 | − | − | 40/62 | 5 | 15 | 18 |

positive prick tests. This stratification graded patients with unequivocal atopy through to those with least evidence of atopy.

*Prognostic factors affecting survival in HD*

These are shown in Table II. The known prognostic factors can be divided into 2 types. First, those like age and sex which precede and are separate from the disease process but which somehow modify the disease. Second, those factors like stage, symptoms and histology which are expressions of the severity of the disease. The reasons for the degree of severity in an individual patient are unknown.

**Table II** Statistical analysis of prognostic factors for survival in Hodgkin's disease

| *Prognostic factor* | | *Relative death rate (O/E)* | $\chi^2$ *for trend* | *P-value* |
|---|---|---|---|---|
| *Stage* | I | 0.33 | | |
| | II | 0.44 | | |
| | III | 1.22 | 8.67 | 0.003 |
| | IV | 1.56 | | |
| *Symptoms* | A | 0.54 | 14.12 | 0.0002 |
| | B | 1.83 | | |
| *Histology* | LP | 0.19 | | |
| | NS | 0.66 | 15.63 | 0.0001 |
| | MC | 0.9 | | |
| | LD | 2.7 | | |
| *Age* | <40 yr. | 0.5 | 16.77 | <0.0001 |
| | ≥40 yr. | 1.9 | | |
| *Sex* | Male | 1.17 | 1.28 | 0.3 |
| | Female | 0.76 | | |
| *Atopic Status* | Group 1 | 0.22 | | |
| | Group 2 | 0.38 | | |
| | Group 3 | 0.85 | 18.24 | <0.0001 |
| | Group 4 | 1.89 | | |

The widely described effect of stage, symptoms and histology on prognosis was again confirmed by this study. Less often reported is the influence of age on survival (Axtell *et al.*, 1972) which has disclosed a markedly decreased survival in older patients. The age analysis shown in Table II divided patients into those above and below 40 years of age because this corresponded with the trough between the 2 peaks of the bimodal age distribution in HD. However, a number of age strata were analysed and all showed an adverse prognosis in age groups >40 years, and the prognosis was worse the older the age grouping. The relative death rates for the 2 age groups were little changed and still significant after stratification for stage ($P=0.0001$), symptoms ($P=0.0001$) and histology ($P=0.0006$).

Females had a slightly improved survival compared with males but this was not significant.

The novel discovery in this study was the significant effect of atopic stratification related to survival in HD (Figure 1). When survival of these

**Figure 1** Survival in HD based on atopic stratification, No. of patients alive and under observation at diagnosis and 12 monthly therafter. Group 1: 29, 24, 20, 15, 13, 8 & 7. Group 2: 26, 23, 19, 11, 8, 4 & 2. Group 3: 31, 21, 17, 12, 11, 5 & 1. Group 4: 62, 40, 25, 21, 16, 9 & 4.

groups was analysed (Table II) there was a highly significant trend whereby survival was ranked Group 1 > 2 > 3 > 4.

*Distribution of the other prognostic factors among the atopic strata*

The difference in survival between Groups 1–4 could be due to less severe disease in Groups 1 and 2 (Stage I or II, A symptom status and LP or NS histology) with correspondingly more severe disease in Groups 3 and 4 (Stage III or IV, B symptom status and MC or LD histology). There was, in fact, significant heterogeneity among the groups both as far as stage and symptoms (Table III) and histology (Table IV) were concerned, but not in the clear cut manner outlined above. In Group 1 there was an increased proportion of patients with Stage IA and IIA disease. There was a concomitant decrease in patients with Stages IB–IVB. The reverse was found in Group 3. As there were only 4 patients in Stage IB and IIB these stages were grouped with IIIB and IVB. The heterogeneity of histology among the atopic strata is shown in Table IV. The high proportion of patients in Group 3 with NS histology was expected from a previous study (Amlot & Green, 1978). Group 4 had an increase of LD histology.

Group 4 also had an older mean age than the other 3 atopic groups. The mean age (and age range) were as follows: Group 1, 35.8 y. (15–73); Group 2, 31.8 y (13–59); Group 3, 34.4 y (16–66); Group 4, 44.8 y (17–81).

**Table III** Distribution of stage and symptoms among the atopic groups

| Atopic group | Stage and symptom status: IA, IIA | IIIA, IVA | IB–IVB |
|---|---|---|---|
| 1 | 19 (65.5%) | 7 (24.1%) | 3 (10.4%) |
| 2 | 7 (26.9%) | 10 (38.5%) | 9 (34.6%) |
| 3 | 3 (9.7%) | 7 (22.6%) | 21 (67.7%) |
| 4 | 19 (30.6%) | 14 (22.6%) | 29 (46.8%) |
| ALL | 48 (32.4%) | 38 (25.7%) | 62 (41.9%) |

$\chi^2 = 29.59$ ($P = 0.00005$)

**Table IV** Distribution of histology among the atopic groups

| Atopic group | Histology: LP | NS | MC | LD |
|---|---|---|---|---|
| 1 | 6 (20.7%) | 8 (27.6%) | 10 (34.5%) | 5 (17.2%) |
| 2 | 1 (3.9%) | 7 (26.9%) | 14 (53.8%) | 4 (15.4%) |
| 3 | 3 (9.7%) | 17 (54.8%) | 9 (29%) | 2 (6.5%) |
| 4 | 8 (12.9%) | 10 (16.1%) | 27 (43.6%) | 17 (27.4%) |
| ALL | 18 (12.2%) | 42 (28.4%) | 60 (40.5%) | 28 (18.9%) |

$\chi^2 = 22.03$ ($P = 0.001$)

*Analysis of atopic strata allowing for other prognostic factors*

The atopic groups differed as regards age, stage, symptoms and histology and since the relative death rates varied with each of these prognostic factors (Table II) their contribution to the effect of atopy on survival had to be assessed. Allowance for each of these prognostic factors and the adjusted relative death rates are shown in Table V. The trend for the atopic groups remains highly significant. However the effect seen may still have been due to a combination of prognostic factors so the atopic groups had to be compared after retrospective stratification with respect to more than one of these factors using the logrank method. The combination of factors chosen was age, symptoms and histology as each showed an effect

**Table V** Adjusted relative death rates allowing for age (<40 y; ≥40 y), stage symptoms and histology individually.

| Atopic strata | Adjusted relative death rates (O/E) allowing for: Age | Stage | Symptoms | Histology |
|---|---|---|---|---|
| Group 1 | 0.2 | 0.31 | 0.34 | 0.25 |
| Group 2 | 0.52 | 0.38 | 0.38 | 0.33 |
| Group 3 | 1.01 | 0.68 | 0.62 | 1.09 |
| Group 4 | 1.61 | 1.84 | 1.86 | 1.7 |
| $\chi^2$ for trend = | 13.87 | 15.01 | 14.96 | 16.2 |
| $P$ = | 0.0002 | 0.0001 | 0.0001 | 0.0001 |

upon prognosis independent of the others. Age, as mentioned above, was an important factor even after stratification for each of the other prognostic factors individually. Hence, age must be allowed for when comparing the atopic groups. However, with stage the relative death rates did not vary significantly after stratification for symptoms whereas the death rates varied significantly between those with A (O/E=0.65) and B (O/E=1.40) symptoms after stratification for stage ($P$=0.02). Thus if allowance is made for the effect symptoms have on prognosis there is no need to make allowance for the effect of stage as well. Symptoms were an important factor after allowance for age ($P$=0.0002) and histology (0.01). Histology was important after allowance for symptoms ($P$=0.002) and age ($P$=0.001).

The atopic groups were compared after stratification with respect to age (<40 y, ≥40 y), symptoms and histology (Table VI). There was still a significant trend ($P$=0.004) in the adjusted relative death rates from 0.37 in Group 1 to 1.36 in Group 4. Clearly the patients of Group 4 differed from those of the other 3 and those in groups 1 and 2 were very similar both in their relative death rates (Tables V and VI) and in having a history of atopic symptoms either in themselves or in their families. Group 3 patients had very high IgE levels but no atopy and their relative death rate was closer to those of Groups 1 and 2 and it could be shown that the relative death rates for Groups 1, 2 and 3 did not differ significantly ($P$=0.4). On the other hand Group 4 had a significantly worse prognosis than groups 1, 2 and 3 combined after stratification for age symptoms and histology ($P$=0.03).

Patients ≥60 y had the worst survival and group 4 had a much higher percentage of these older patients than the other groups. The stratification for age in the previous analysis was for those <40 y or ≥40 y. In view of the number of subgroups already in the analysis and that the atopic groups 1, 2 and 3 had 3 or less aged 60 or over, it was undesirable to repeat the previous analysis with the ≥40 y group split into 40–59 y and 60 y. However the analysis was repeated excluding those ≥60 y. The relative death rates were very similar to those of the preceeding analysis and are shown in Table VI.

**Table VI** Adjusted relative death rates after retrospective stratification for age (<40 y, ≥40 y), symptoms and histology.

| *Atopic group* | *Adjusted relative death rates (O/E)* *All ages* | | *Age<60 yr.* |
|---|---|---|---|
| 1 | 0.37 | 0.45 | |
| 2 | 0.54 | | 0.60 |
| 3 | 0.84 | | 0.74 |
| 4 | 1.36 | | 1.39 |
| $\chi^2$ for trend | 8.43 | | 4.94 |
| $P$ | 0.004 | | 0.03 |

*Cause of death*

In the majority of deaths the primary cause was HD and there was no clear difference in cause of death between the atopic strata. Complicated deaths from other causes were: in Group 1, one patient with paraplegia had renal infection and recurrent septicaemia and another had a hypereosinophilic state (50,000 $\mu l^{-1}$) followed by acute tubular necrosis subsequent to laparotomy while both were in relapse. In Group 2 one patient died of acute myeloid leukaemia while in CR. In Group 3 three patients died in CR, one from myocardial infarction, and the other two from carcinoma of the lung. In Group 4 one patient died from peritonitis while in CR, one from complications of paraplegia while in relapse, one from multiple system failure with minimal hepatic HD and one patient who died at home while in relapse.

Excluding the patients above re-analysis of survival still showed a highly significant trend ($P$<0.0001) for atopic strata.

### Discussion

Atopic status was stratified in this study because it is known to be a multifactorial disease often having a late onset. Thus apart from those patients who had clearly suffered from atopic symptoms prior to developing HD (Group 1) there was a group of patients with a genetic predisposition to atopy many of whom had evidence of atopic hypersensitivity without having developed atopic symptoms (Group 2). The peak age for subjects developing hay fever lies, like HD, in the third decade and its development is more prevalent in those with a family history of atopy. Even in those Groups 3 and 4 where atopy was not evident there were occasional individuals with positive prick tests and who therefore risked development of atopic symptoms at a later date. The relationship between prolonged survival and positive prick tests will be dealt with in a subsequent paper. Comparison of survival, prompted initially by the observation that atopic patients (Group 1) rarely had "B" symptoms, led to the discovery that this atopic stratification was associated with survival whereby atopy had a favourable effect even after stratification for age, symptoms and histology.

There is a difference between prognostic factors such as age, sex and atopy coming before the onset of HD and those such as stage, symptoms and histology which are established with its diagnosis. The latter group are clearly the most reliable in predicting prognosis for HD but it leaves unanswered the question as to why in an individual patient HD presents with a lesser or greater stage and severity. Length of history is not a reliable guide, even assuming that the disease progresses at a uniform rate in all patients. The site at which disease starts may be a more important indicator since abdominal lymphadenopathy is frequently associated with "B" symptoms and evidently can progress much further before being detected than in patients presenting with peripheral lymphadenopathy in the neck. Histological classification provides suggestive evidence that lymphocytes and "benign" histiocytes represent a host response to Reed-Sternberg (RS) and Hodgkin's cells ("malignant histiocytes") and thus are a key factor in restricting the spread of HD (Lukes & Butler, 1966; Coppleson *et al.,* 1973). Although there is clear evidence that the higher the ratio of lymphocytes and "benign" histiocytes to Hodgkin's cells the better the prognosis, no direct evidence of lymphocyte cytotoxicity towards Hodgkin's or RS cells has been forthcoming. This work has been hampered by the difficulty in isolating and establishing tissue culture lines of Hodgkin's cells against which lymphocytes could be tested.

Interest in factors preceding the development of HD yet affecting its final outcome lies in the possibility of identifying host resistance mechanisms for HD. Age of onset is clearly an important factor. Although the effect of age is independent of the other prognostic variables, among 19 in the older age group ($\geq 60$ y) there was a 95% frequency of MC or LD histology and a 63% frequency of Stage IIIB or IVB. These findings were similar to the same age group reported by Lokich *et al.,* (1974) in which 83% of 47 patients had MC or LD histology and 81% had stage III or IV disease. Median survival of 5 months was very poor and may partly have been explained by the palliative treatment given in some cases. We attempted to give full treatment in this group and still only achieved a median survival of 10 months for the 12 who had died during the study period. Older patients tolerated full dosage of treatment poorly and prolonged bouts of myelosuppression with its associated complications probably played a significant part in the poor results. Evidently age *per se* is not a manipulable resistance factor and the lack of resistance to HD in the $\geq 60$ y old patients does not define the causative factors.

The recognition of atopy as having a favourable effect upon survival in HD raises interest in the possible mechanism by which it acts. Atopy is a complex disorder involving immunological mechanisms of immediate hypersensitivity as well as non-immunological ones such as increased sensitivity to $\alpha$ adrenergic stimuli and decreased $\beta$ adrenergic receptors. However, it is the vigorous production of IgE antibodies in response to low doses of antigen that is the most clearly defined feature. Consequently the protective mechanism most readily suggested is the production of anti-tumour IgE antibody and limitation of disease spread as a result of tissue sensitisation and the action of accessory effector cells. It is known that rodent melanoma and fibrosarcoma induce specific reaginic responses (Bartholomaeus & Kaest, 1972; Broom & Alexander, 1975). Furthermore there have been suggestions of both basophil (Dvorak *et al.,* 1973) and mast cell (Likhite, 1974) involvement in tumour resistance. Against this we have been unable to demonstrate IgE antibody bound to Hodgkin's cells in fresh biopsies or subsequent binding using high IgE serum (unpublished results) but the method used (immunofluorescence) may not have been sensitive enough. Also reports of wheal and flare responses to tumour extracts are rare (Grace & Kondo, 1958) in contrast to those frequently reported in helminthic disease.

A less obvious means by which atopy may influence tumour growth is based on the cellular mechanisms underlying control IgE antibody production. It is not surprising that the potentially damaging IgE antibody is under strong T lymphocyte suppressor control which is presumably abnormal in atopic subjects. Helminthic parasites can stimulate IgE synthesis with loss of suppressor control during infestation. It was found that tumour growth is inhibited in rodents infested with helminths (Keller *et al.,* 1971). In these experiments no reaginic antitumour antibody was demonstrated but surprisingly the tumour inhibition was abolished when rodents were treated with anti-lymphocyte antiserum. At the time it was difficult to interpret these results but development of specific antisera for rodent supressor cells has demonstrated that tumour growth may be inhibited when suppressor cells are eradicated (Perry *et al.,* 1978; Tilkin *et al.,* 1981). These studies suggest that in normal individuals the powerful suppressor control of IgE synthesis may be linked to suppressor control of cellular responses towards weak antigenic systems such as tumour antigens. Abrogation of suppressor control may paradoxically benefit the tumour bearing host. It may also be relevant that in both these experimental systems tumour growth was inhibited but not abolished which is similar to the effect of atopy in HD.

HD is a variable disease that runs a variable course. Assuming that it is a single disease entity and that its different manifestations and progression are reflections of a "host-tumour interaction" then factors that influence it are of interest for their positive and negative effects on prognosis. This study shows that atopy is a factor that precedes the development of HD and correlates favourably with its outcome.

We are grateful to Dr. G.A.K. Missen and Dr. D.R. Turner for histological classification at Guy's Hospital and the BNLI panel for reporting on tissue at the London Hospital. The following patiently provided follow up information on patients—Dr. E. Wilson and Dr. M. Minton (London Hospital), Dr. D. Barrett (Pembury Hospital), Dr. I. Roberts (Kent & Canterbury Hospital), Dr. E.L. Offerman (Queen Mary's Hospital), Dr. G.P. Deutsch (Royal Sussex Hospital) and Dr. T.J. Mott (Ipswich Hospital). Prick tests were kindly performed by P. Parkes, and W. Henderson organised the family questionnaires.

This study was supported by a grant from the Cancer Research Campaign.

**References**

AMLOT, P.L. & GREEN, L. (1978). Atopy and immunoglobulin E concentrations in Hodgkin's disease and other lymphomas. *Br. Med. J.*, **i**, 327.

AMLOT, P.L. & SLANEY, J.M. (1981). Hypergammaglobulinaemia E in Hodgkin's disease and its relationship to atopy or a familial predisposition to atopy. *Int. Arch. Allergy Appl. Immunol.*, **64**, 138.

AXTELL, L.M., MYERS, M.H., THOMAS, L.H., BERARD, C.W., KAGAN, A.R. & NEWELL, G.R. (1972). Prognostic indicators in Hodgkin's disease. *Cancer*, **29**, 1481.

BARTHOLOMAEUS, W.N., & KAEST, D. (1972). Reaginic antibody to tumour and alloantigens in mice. *Nature*, (New Biol.) **239**, 206.

BROOM, B.C. & ALEXANDER, P. (1975). Rat tumour allografts evoke anaphylactic antibody responses. *Immunology*, **28**, 1033.

CARBONE, P.P., KAPLAN, H.S., MUSSHOFF, K., SMITHERS, D.W. & TUBIANA, M. (1971). Report of the committee on Hodgkin's disease staging classification. *Cancer Res.*, **31**, 1860.

COCA, A.F. & COOKE, R.A. (1923). On the classification of the phenomena of hypersensitiveness. *J. Immunol.*, **8**, 163.

COPPLESON, L.W., RAPPAPORT, H., STRUM, S.B. & ROSE, J. (1973). Analysis of the Rye Classification of Hodgkin's disease. The prognostic significance of cellular composition. *J. Natl Cancer Inst.*, **51**, 379.

DE VITA, V.T., SERPICK, A.A. & CARBONE, P.P. (1970). Combination chemotherapy in the treatment of advanced Hodgkin's disease. *Ann. Intern. Med.*, **73**, 881.

DVORAK, H.F., DVORAK, A.M. & CHURCHILL, W.H. (1973). Immunologic rejection of diethylnitrosamine-induced hepatomas in strain 2 guinea pigs. *J. Exp. Med.*, **137**, 751.

DWORIN, M., DIAMOND, H.D. & CRAVER, L.F. (1955). Hodgkin's disease and allergy. *Cancer*, **8**, 128.

FISHERMAN, E.W. (1960). Does the allergic diathesis influence malignancy? *J. Allergy*, **31**, 74.

GRACE, J.T. & KONDO, T. (1958). Investigations of host resistance in cancer patients. *Ann. Surg.*, **148**, 631.

KAPLAN, H.S. & ROSENBERG, S.A. (1975). The management of Hodgkin's disease. *Cancer*, **36**, 796.

KELLER, R., OGILVIE, B.M. & SIMPSON, E., (1971). Tumour growth in nematode infected animals. *Lancet*, **i**, 678.

LIKHITE, V.V. (1974). The delayed and lasting rejection of mammary adenocarcinoma cell tumours in DBA/2 mice with use of killed Bordetella pertussis. *Cancer Res.*, **34**, 1027.

LOKICH, J.J., PINKUS, G.S. & MAHONEY, W.C. (1974). Hodgkin's disease in the elderly. *Oncology*, **19**, 484.

LUKES, R.J. & BUTLER, J.J. (1966). The pathology and nomenclature of Hodgkin's disease. *Cancer Res.*, **26**, 1063.

MACKAY, W.D. (1966). The incidence of allergic disorders and cancer. *Br. J. Cancer*, **20**, 434.

McCORMICK, D.P., AMMAN, A.J., ISHIZAKA, K., MILLER, D.G. & HONG, R. (1971). A study of allergy in patients with malignant lymphoma and chronic lymphocytic leukaemia. *Cancer*, **27**, 93.

McKEE, W.D., ARNOLD, C.A. & PERLMAN, M.D. (1967). A double blind study of the comparative incidence of malignancy and allergy. *J. Allergy*, **39**, 394.

NICHOLSON, W.M., BEARD, M.E.J., CROWTHER, D. & 5 others. (1970). Combination chemotherapy in generalised Hodgkin's disease. *Br. Med. J.*, **iii**, 7.

OGILVIE, B.M. & JONES, V.E. (1973). Immunity in the parasitic relationship between helminths and hosts. *Prog. Allergy*, **17**, 93.

PERRY, L.L., BENACERRAF, B., McCLUSKY, R.T. & GREENE, M.I. (1978). Enhanced syngeneic tumour destruction by *in vivo* inhibition of suppressor *T* cells using anti-IJ alloantiserum. *Am. J. Pathol.*, **92**, 491.

PETO, R., PIKE, M.C., ARMITAGE, P. & 7 others. (1977). Design and analysis of randomised clinical trials requiring prolonged observation of each patient. *Br. J. Cancer*, **35**, 1.

ROSENBAUM, J.T. & DWYER, J.M. (1977). the role of IgE in the immune response to neoplasia: A review. *Cancer*, **39**, 11.

SHAPIRO, S. HEINONEN, O.P. & SISKING, U. (1971). Cancer and allergy. *Cancer*, **28**, 396.

TILKIN, A.F., SCHAAF-LAFONTAINE, N., VAN ACKER, A., BOCCADORO, M. & URBAIN, J. (1981). Reduced tumour growth after low dose irradiation or immunisation against blastic suppressor cells. *Proc. Natl Acad. Sci.*, **78**, 1809.

WALDMAN, T.A., BULL, J.M., BRUCE, R.M. & 4 others. (1974). Serum immunoglobulin *E* levels in patients with neoplastic disease. *J. Immunol.*, **113**, 379.


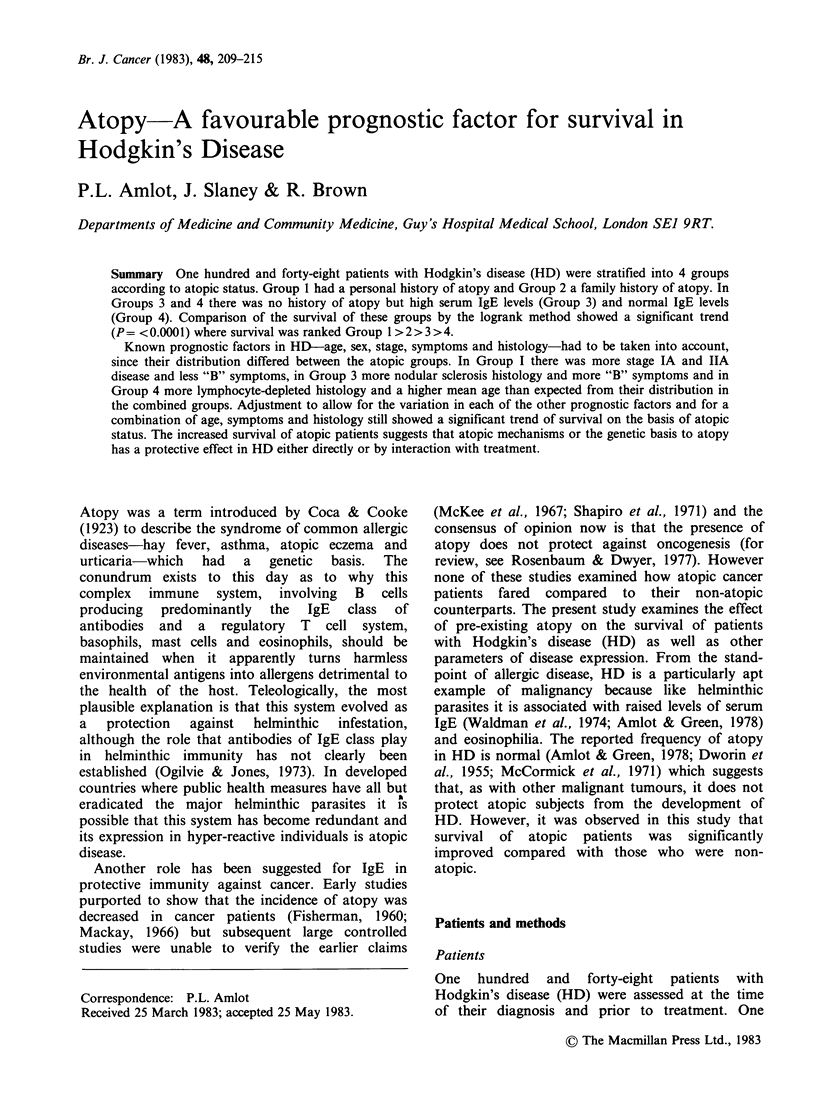

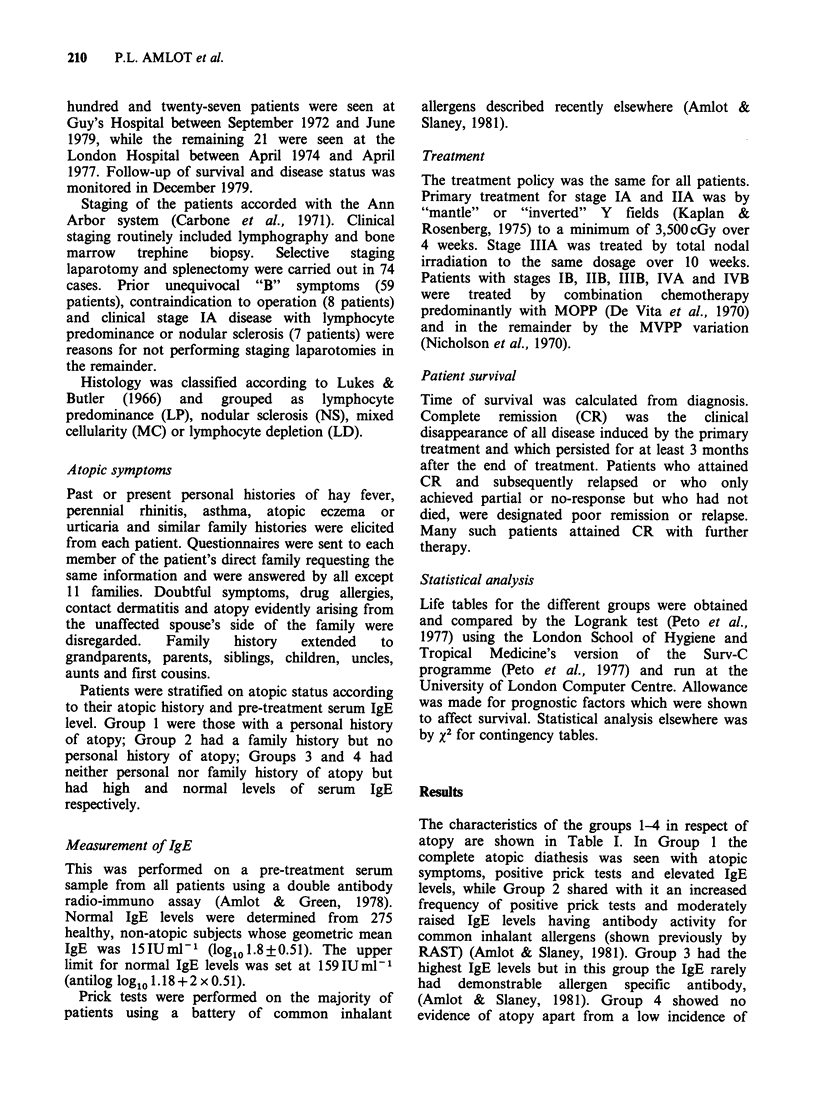

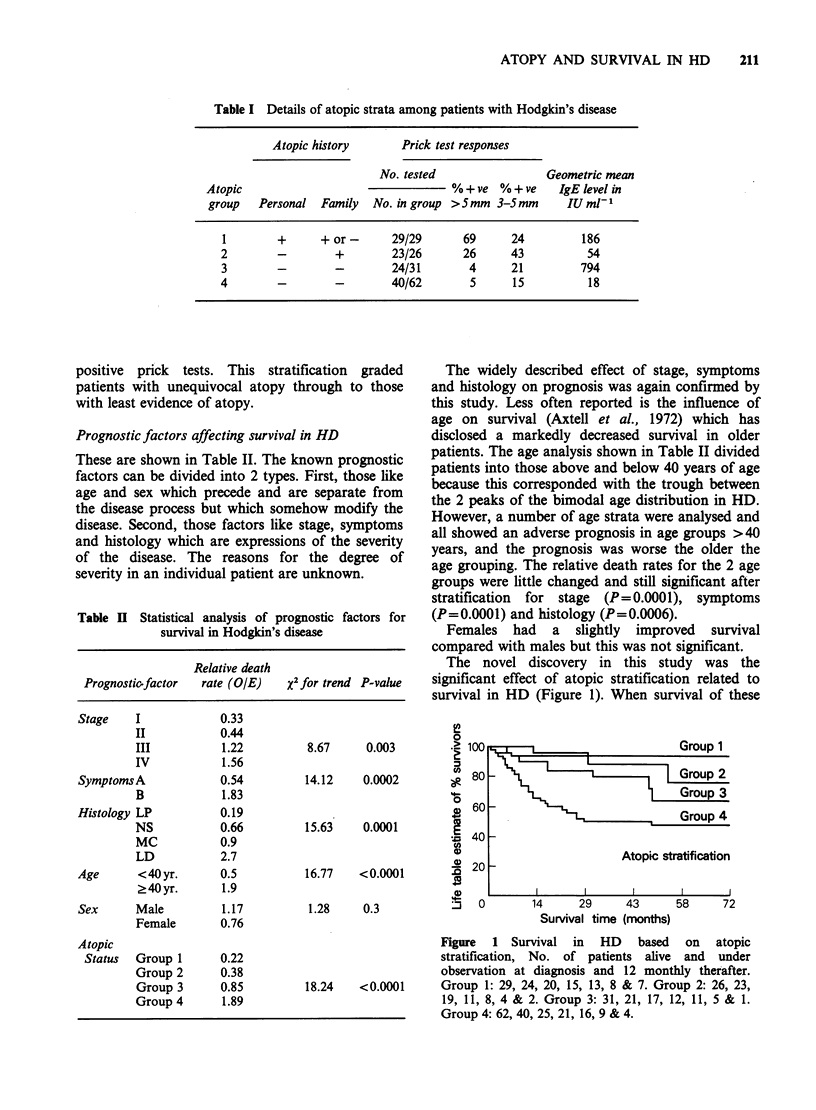

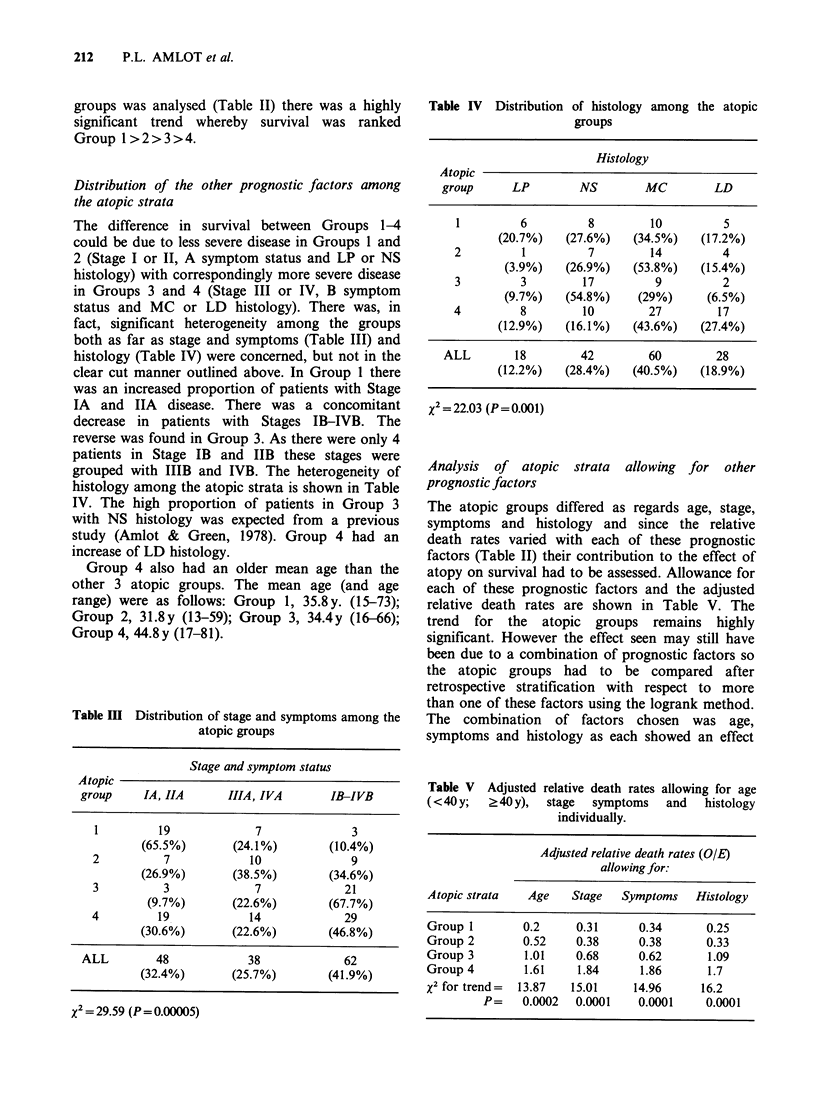

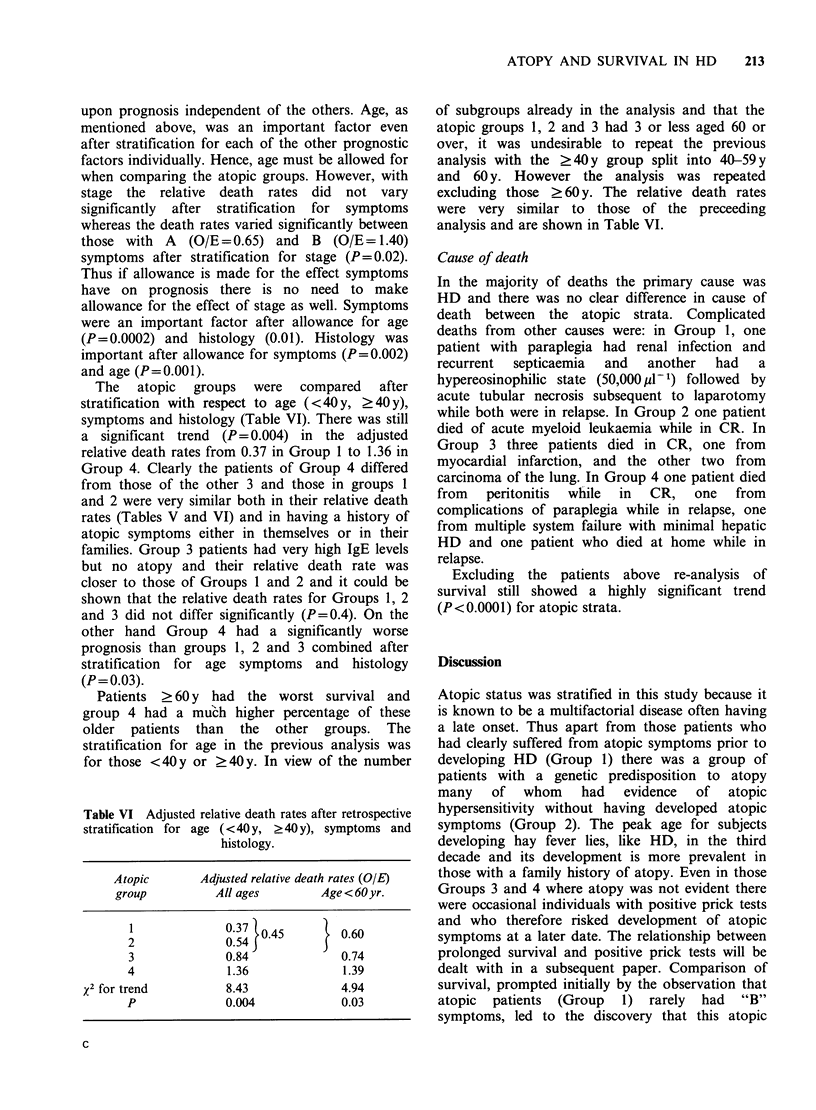

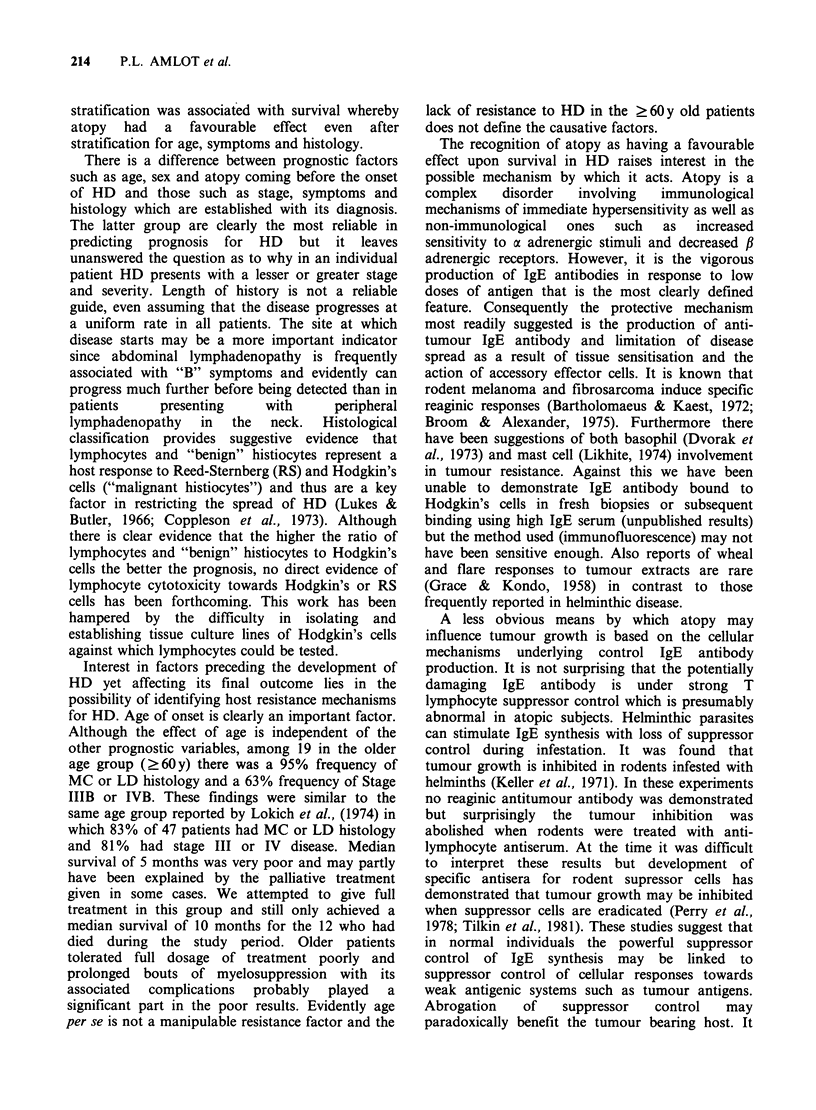

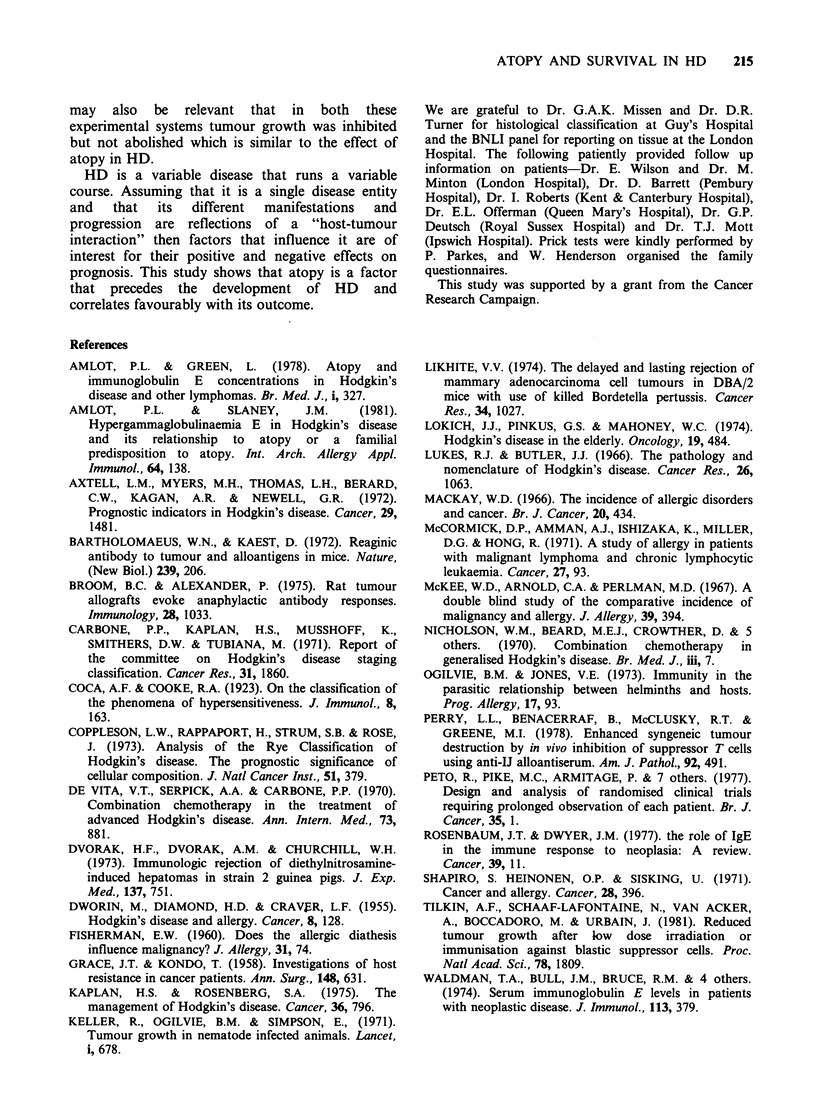


## References

[OCR_00760] Amlot P. L., Green L. A. (1978). Atopy and immunoglobulin E concentrations in Hodgkin's disease and other lymphomas.. Br Med J.

[OCR_00765] Amlot P. L., Slaney J. (1981). Hypergammaglobulinaemia E in HOdgkin's disease and its relationship to atopy or a familial predisposition to atopy.. Int Arch Allergy Appl Immunol.

[OCR_00772] Axtell L. M., Myers M. H., Thomas L. H., Berard C. W., Kagan A. R., Newell G. R. (1972). Prognostic indicators in Hodgkin's disease.. Cancer.

[OCR_00778] Bartholomaeus W. N., Keast D. (1972). Reaginic antibody to tumour and alloantigens in mice.. Nat New Biol.

[OCR_00783] Broom B. C., Alexander P. (1975). Rat tumour allografts evoke anaphylactic antibody responses.. Immunology.

[OCR_00788] Carbone P. P., Kaplan H. S., Musshoff K., Smithers D. W., Tubiana M. (1971). Report of the Committee on Hodgkin's Disease Staging Classification.. Cancer Res.

[OCR_00799] Coppleson L. W., Rappaport H., Strum S. B., Rose J. (1973). Analysis of the Rye classification of Hodgkin's disease. The prognostic significance of cellular composition.. J Natl Cancer Inst.

[OCR_00817] DWORIN M., DIAMOND H. D., CRAVER L. F. (1955). Hodgkin's disease and allergy.. Cancer.

[OCR_00805] Devita V. T., Serpick A. A., Carbone P. P. (1970). Combination chemotherapy in the treatment of advanced Hodgkin's disease.. Ann Intern Med.

[OCR_00811] Dvorak H. F., Dvorak A. M., Churchill W. H. (1973). Immunologic rejection of diethylnitrosamine-induced hepatomas in strain 2 guinea pigs: participation of basophilic leukocytes and macrophage aggregates.. J Exp Med.

[OCR_00821] FISHERMAN E. W. (1960). Does the allergic diathesis influence malignancy?. J Allergy.

[OCR_00829] Kaplan H. S., Rosenberg S. A. (1975). The management of Hodgkin's disease.. Cancer.

[OCR_00838] Likhite V. V. (1974). The delayed and lasting rejection of mammary adenocarcinoma cell tumors in DBA-2 mice with use of killed Bordetella pertussis.. Cancer Res.

[OCR_00844] Lokich J. J., Pinkus G. S., Moloney W. C. (1974). Hodgkin's disease in the elderly.. Oncology.

[OCR_00848] Lukes R. J., Butler J. J. (1966). The pathology and nomenclature of Hodgkin's disease.. Cancer Res.

[OCR_00853] Mackay W. D. (1966). The incidence of allergic disorders and cancer.. Br J Cancer.

[OCR_00857] McCormick D. P., Ammann A. J., Ishizaka K., Miller D. G., Hong R. (1971). A study of allergy in patients with malignant lymphoma and chronic lymphocytic leukemia.. Cancer.

[OCR_00873] Ogilvie B. M., Jones V. E. (1973). Immunity in the parasitic relationship between helminths and hosts.. Prog Allergy.

[OCR_00833] Ogilvie B. M., Simpson E., Keller R. (1971). Tumour growth in nematode-infected animals.. Lancet.

[OCR_00878] Perry L. L., Benacerraf B., McCluskey R. T., Greene M. I. (1978). Enhanced syngeneic tumor destruction by in vivo inhibition of suppressor T cells using anti-I-J alloantiserum.. Am J Pathol.

[OCR_00895] Shapiro S., Heinonen O. P., Siskind V. (1971). Cancer and allergy.. Cancer.

[OCR_00899] Tilkin A. F., Schaaf-Lafontaine N., Van Acker A., Boccadoro M., Urbain J. (1981). Reduced tumor growth after low-dose irradiation or immunization against blastic suppressor T cells.. Proc Natl Acad Sci U S A.

[OCR_00906] Waldmann T. A., Bull J. M., Bruce R. M., Broder S., Jost M. C., Balestra S. T., Suer M. E. (1974). Serum immunoglobulin E levels in patients with neoplastic disease.. J Immunol.

